# Fire suppression and seed dispersal play critical roles in the establishment of tropical forest tree species in southeastern Africa

**DOI:** 10.1038/s41598-021-95752-7

**Published:** 2021-08-12

**Authors:** Tomohiro Fujita

**Affiliations:** Center for Climate Change Adaption, National Institute for Environmental Studies, 16-2, Onogawa, Tsukuba, Ibaraki 305-8506 Japan

**Keywords:** Ecology, Plant sciences

## Abstract

This study examined the mechanisms of facilitation and importance of seed dispersal during establishment of forest tree species in an Afrotropical woodland. Seedling survival of *Syzygium guineense* ssp. *afromontanum* was monitored for 2.5 years at four different microsites in savannah woodland in Malawi (southeastern Africa) under *Ficus natalensis* (a potential nurse plant), *Brachystegia floribunda* (a woodland tree), *Uapaca kirkiana* (a woodland tree), and at a treeless site. The number of naturally established forest tree seedlings in the woodland was also counted. Additionally, *S. guineense* ssp. *afromontanum* seed deposition was monitored at the four microsites. Insect damage (9% of the total cause of mortality) and trampling by ungulates (1%) had limited impact on seedling survival in this area. Fire (43%) was found to be the most important cause of seedling mortality and fire induced mortality was especially high under *U*. *kirkiana* (74%) and at treeless site (51%). The rate was comparatively low under *F. natalensis* (4%) and *B. floribunda* (23%), where fire is thought to be inhibited due to the lack of light-demanding C4 grasses. Consequently, seedling survival under *F*. *natalensis* and *B*. *floribunda* was higher compared with the other two microsites. The seedling survival rate was similar under *F*. *natalensis* (57%) and *B*. *floribunda* (59%). However, only a few *S*. *guineense* ssp. *afromontanum* seedlings naturally established under *B*. *floribunda* (25/285) whereas many seedlings established under *F*. *natalensis* (146/285). These findings indicate that the facilitative mechanism of fire suppression is not the only factor affecting establishment. The seed deposition investigation revealed that most of the seeds (85%) were deposited under *F. natalensis*. As such, these findings suggest that in addition to fire suppression, dispersal limitations also play a role in forest-savannah dynamics in this region, especially at the community level.

## Introduction

Tropical forests and savannah-woodlands are major terrestrial biomes in tropical landscapes^1^. Although their global occurrence is controlled by climate, on a regional to local scale, these two contrasting vegetation types occur within identical climatic conditions^2,3^. Recent studies have reported the expansion of tropical forests into adjacent savannah-woodlands in many parts of the world^4–7^. These expansions depend on the successful establishment of forest tree species because many constraints limit their establishment in savannah-woodland, including seed- and establishment-limitation^8^ and seed predation^9^. Historically, more emphasis has been placed on the importance of plant–plant interactions (i.e., facilitation) during the recruitment of forest tree species in savannah-woodland^10–15^. Facilitation has been shown to exert both a direct and indirect effect on establishment through the modification of abiotic and biotic conditions by particular so-called nurse plants^16,17^.

Previous studies suggest that seedling abundance and the survival of forest tree species under nurse plants is higher than in treeless areas of savannah-woodland^10–12^. These studies further suggest that the facilitative effects, such as amelioration of water stress act by suppressing the occurrence of fire and improving soil properties. However, the specific mechanisms remain unknown particularly in African savannah-woodland, because studies on the relative importance of different factors affecting seedling survival are limited there.

In addition to the facilitative effect on seedling survival, seed dispersal is thought to be critical for the recruitment of forest tree species in savannah-woodland. Indeed, if seeds of forest tree species are not dispersed, facilitation of seedling survival is not even a factor. Thus, examination of seed dispersal should be considered in investigations of forest tree recruitment in savannah-woodland.

This study attempted to clarify the mechanisms of forest tree recruitment in an Afrotropical woodland in northern Malawi, southeastern Africa. *Ficus natalensis* (Moraceae) was included as a nurse plant because trees in this genus are widely known to play a role in establishment of tropical savannah-woodlands and post-agricultural sites^18,19^. In some cases, *Ficus* trees drive the creation of forest patches, called “nucleation”^20^. In northern Malawi, the circular forest patches often occur within woodlands, with large freshy fruit trees, such as *Ficus natalensis*, existing in the center^21^. These structures are a general feature of “nucleated forest patches” rather than fragmented forests^5,22^.

In this study, the seedling survival of *Syzygium guineense* ssp. *afromontanum* (a common forest tree species) was monitored under *F. natalensis*, *Brachystegia floribunda* (a woodland tree), *Uapaca kirkiana* (a woodland tree), and at a treeless site for 2.5 years. During monitoring, the cause of seedling mortality was also recorded. Using the obtained data, this study attempted to determine the specific mechanisms of the facilitative effect on forest tree establishment in an Afrotropical woodland. The number of forest tree seedlings in the woodland was also quantified in order to confirm the natural establishment of forest tree species there. In addition, the seed rain of *S. guineense* ssp. *afromontanum* at the four microsites was monitored to take into account processes other than facilitation in the recruitment of forest tree species. Overall, this study aimed to determine the following: (1) the most important facilitative mechanism affecting the seedling survival of *S. guineense* ssp. *afromontanum*, and (2) whether only the facilitative mechanism can explain the recruitment of forest tree species in Afrotropical woodland or whether other processes such as seed dispersal might affect them.

## Materials and methods

### Study area

This study was conducted in northern Malawi in southeastern Africa, where Afrotropical woodlands, known as miombo woodland, covers approximately 2.7 million km^2^. Miombo woodland is composed of three closely related genera of Caesalpiniaceae: *Brachystegia*, *Julbernardia*, and *Isoberlinia*^23^. These are largely deciduous trees that reach a canopy height of 10–20 m. Miombo woodland is also characterized by a continuous C4 grass layer. Montane rainforests also occur in this region on mountain crests and in valleys^24^ and in contrast are composed of evergreen trees with a tall canopy (20–25 m) and numerous lianas.

The study site (10°58′S, 34°04′E) was situated in a local zone governed by a rural village. Mean annual precipitation is more than 1200 mm on the north Vipya Plateau^25^. Miombo woodland is predominant in the area but some montane rainforests also occur on mountain crests (> 1800 m asl) and in valleys. Besides, circular forest patches (~ 10–1800 m^2^; hereafter referred to as forest patches) montane rainforest tree species are also found within the miombo woodland (1700–1800 m asl^21^). In this study site, miombo woodland is burned by locals during late dry season (September to December) approximately every 2–3 years. Fire rarely spreads far into the montane rainforest due to its closed canopy, lack of grass species and humid understory^26^. Antelopes such as the common duiker (*Sylvicapra grimmia*) are often found grazing there, but other large herbivores such as the elephant (*Loxodonta africana*) and African buffalo (*Syncerus caffer*) are not. Local people rarely cut trees from the miombo woodlands and montane rainforests because of their location far from settlements. The materials used and the processes adopted with the studies are in full compliance with the institutional, national and international guidelines and legislation.

### Focal forest species

*S. guineense* ssp. *afromontanum* F. White (Myrtaceae) is an endemic tree species of montane rainforests of the study area. It reaches heights of 30 m and bears purple berries (fruit size = 1.6 × 1.4 cm, seed size = 1.2 × 1.1 cm, n = 6) during the rainy season (January to March). *S. guineense* ssp. *afromontanum* was selected as the focal species of this study because it is commonly found on the Vipya Plateau^26^ and is common in forest patches within the miombo woodland.

### Characteristics of the studied microsites in the miombo woodland

Four microsites were assessed in the miombo woodland as follows: (1) under *F. natalensis*, (2) under *Brachystegia floribunda*, (3) under *Uapaca kirkiana*, and (4) at treeless sites. *F. natalensis* (Moraceae) is a deciduous tree that reaches heights of 20 m. It occurs primarily in miombo woodland in the region, but is also found in the center of circular forest patches. It has two periods of ripening (August to October and January to April), and produces yellow–red syconia (1.1 × 1.0 cm, n = 10). *B. floribunda* (Caesalpinioideae) is deciduous and reaches heights of 20 m. It is a dominant tree species in the miombo woodlands producing pods from October to January. *U. kirkiana* (Phyllanthaceae) is a smaller tree of up to 13 m and is also a common species in miombo woodland. It bears fleshy fruit (2.6 × 2.6 cm, n = 7) from September to December. The treeless sites consisted of areas in which there were no trees with crowns exceeding a 3-m radius or with a diameter at breast height (dbh) of > 5 cm.

Originally, ten individual replicates were selected for each microsite, but two of the ten replicates were destroyed by locals during the monitoring of seedling survival and seed rain (see below); thus, the results of seedling survival and seed rain were obtained from eight replicates per microsite and the results of environmental variables were from ten replicates.

Eight individual *F*. *natalensis* trees > 50 m from montane rainforest or forest patches (range 56–307 m; mean 169 m) were selected. The mean shortest distance between individuals was 391 m. *B. floribunda*, *U. kirkiana* and the treeless sites were established within 50 m of each *F. natalensis*. Individuals of *B. floribunda* and *U. kirkiana* trees with a height and dbh similar to those of the selected *F. natalensis* were chosen. *F. natalensis*, *B. floribunda* and *U. kirkiana* had little to no canopy overlap with other trees.

### Data collection

#### Environmental variables

Canopy openness and the percentage of grass cover were examined at the each of the four microsites (4 microsites × 10 replicates = 40). To determine canopy cover, four hemispherical canopy photographs were obtained from each replicate during the rainy season (February 2012), after the leaves had fully expanded. Photographs were taken at the mid-point of the crown radius from the trunk or 1 m from the center of the treeless sites in each cardinal direction, at a 1-m height aboveground with a fish-eye lens (Raynox DCR-CF). They were then analyzed using Gap Light Analyzer software^27^ to calculate canopy openness. The percentage of grass cover was estimated visually in four 1 × 1-m quadrats during the dry season (September 2012) before any fires occurred and in the same locations as the canopy photographs were obtained. The overall canopy openness and the percentage of grass cover at each microsite was calculated as the mean of the four direction values.

### *S. guineense* ssp. *afromontanum* seedling survival and causes of mortality

Seeds of *S. guineense* ssp. *afromontanum* were sown in a nursery in January 2012. Four weeks later, the seedlings were transplanted at each of the four microsites. Sixteen seedlings were planted per replicate site in 4 × 4 plant grids spaced 50-cm apart, giving a total of 512 seedlings (16 seedlings × 4 microsites × 8 replicates). All seedlings were marked with a fire-resistant stainless-steel label. They were watered when transplanted, but no additional treatments were applied. One week after transplanting, the seedlings were checked and those that had died due to transplant shock were replaced. Seedling survival and the cause of mortality were then recorded at 1, 6, 7, 10, 19, and 31 months after transplanting. Causes of mortality were determined visually. Drought-induced mortality was determined when the entire seedling became brown and shriveled with no other physical damage (Fig. S1-A). Fire-induced mortality was defined as seedlings that had lost their aboveground parts (only the stainless steel label remaining), plus visual signs of fire damage (Fig. S1-B). Seedlings damaged by insects (notably cutworm [Noctuidae]) were distinguished as those showing insect attack, with a smooth cut close to ground level (Fig. S1-C). These seedlings mostly had the caterpillar’s silk thread. Trampling by ungulates was also classified as a cause of mortality. All causes of mortality not meeting the above criteria were classified as unknown.

### Natural establishment of *S. guineense* ssp. *afromontanum* and other forest tree species in miombo woodland

Four 50 × 50-m (0.25-ha) sites were assessed within the miombo woodland to examine the naturally occurring establishment of forest tree species. Each site included all microsite types. The target species included *S. guineense* ssp. *afromontanum* as well as other forest tree species (Table S1). First, crown projection diagrams were created for each individual tree (dbh > 5 cm) in each of the four sites. The relative proportion of tree cover was then calculated by measuring the crown cover of each tree and summing the area of all tree canopies by species. Then, all the seedlings of forest tree species (0.2–1 m in height) were counted in each sites and their location was recorded (under the tree crown or at the treeless microsite). If a forest tree species was found under a tree crown, the tree crown species was also noted. All individuals were checked for the presence of damage from herbivorous mammals. Observations were conducted in August 2014, before the occurrence of any fires.

### Seed deposition of *S. guineense* ssp. *afromontanum*

Seed deposition of *S. guineense* ssp. *afromontanum* was monitored at each of the four microsites from January to March 2012. Seed traps (70 × 70 cm) made of fine-mesh net and with 5-cm-high sides to prevent seeds from being washed away were installed at ground level in each replicate location. Three seed traps were established at each of the four microsites and the total number of seed traps was 96. The seed traps were located 1 m from the main trunk or 1 m from the center of the treeless site. The direction of the first trap was chosen randomly then the remainder were placed at 120° and 240°, respectively. Each seed trap was visited twice a week and the number of *S. guineense* ssp. *afromontanum* seeds was counted.

### Data analysis

The statistical analyses were done using R software (ver. 2.14.0; R Development Core Team, http://www.r-project.org/). The data on canopy openness and percentage of grass cover did not satisfy the assumption of normality and were therefore analyzed using the Steel–Dwass multiple comparisons test to detect significant differences among microsites. The final percentage of seedling survival was analyzed using a general linear mixed model (GLMM). The analyses assumed binomial distribution and used a logit-link function, including fixed effects of microsite type with random effects of the individual microsite. The number of seed depositions was also analyzed using a GLMM. The analyses assumed Poisson distribution and used a logit-link function, including fixed effects of microsite type with random effects of the individual seed trap. These GLMM were performed using ‘lmer4’ package^28^. Significant differences for seedling survival and the number of seed deposition among the microsites were conducted using Tukey's post hoc test. The post hoc tests were calculated using the glht function in the ‘multicomp’ package^29^.

## Results

### Environmental variables

**Figure 1:**
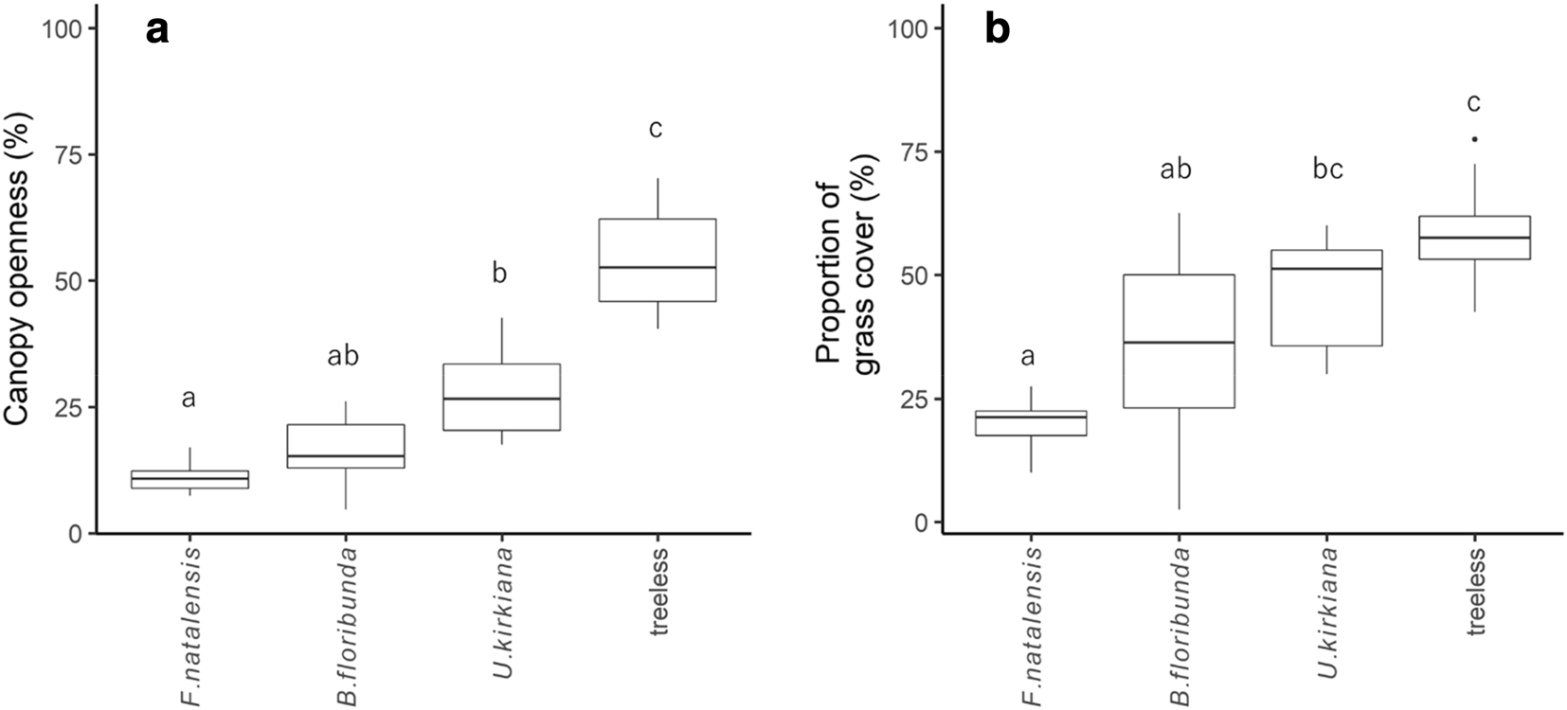
Boxplots of (**a**) canopy openness (n = 40) and (**b**) percentage of grass cover (n = 40) in northern Malawi. All microsites were located in miombo woodlands. Different letters indicate significant differences (*P* < 0.05) among the microsites based on Steel–Dwass post hoc tests.

Canopy openness and the percentage of grass cover were significantly lower under *F. natalensis* and *B. floribunda* compared with the treeless sites (Fig. 1). No differences were observed between the *F. natalensis* and *B. floribunda* sites.

### Seedling survival and causes of mortality

**Figure 2:**
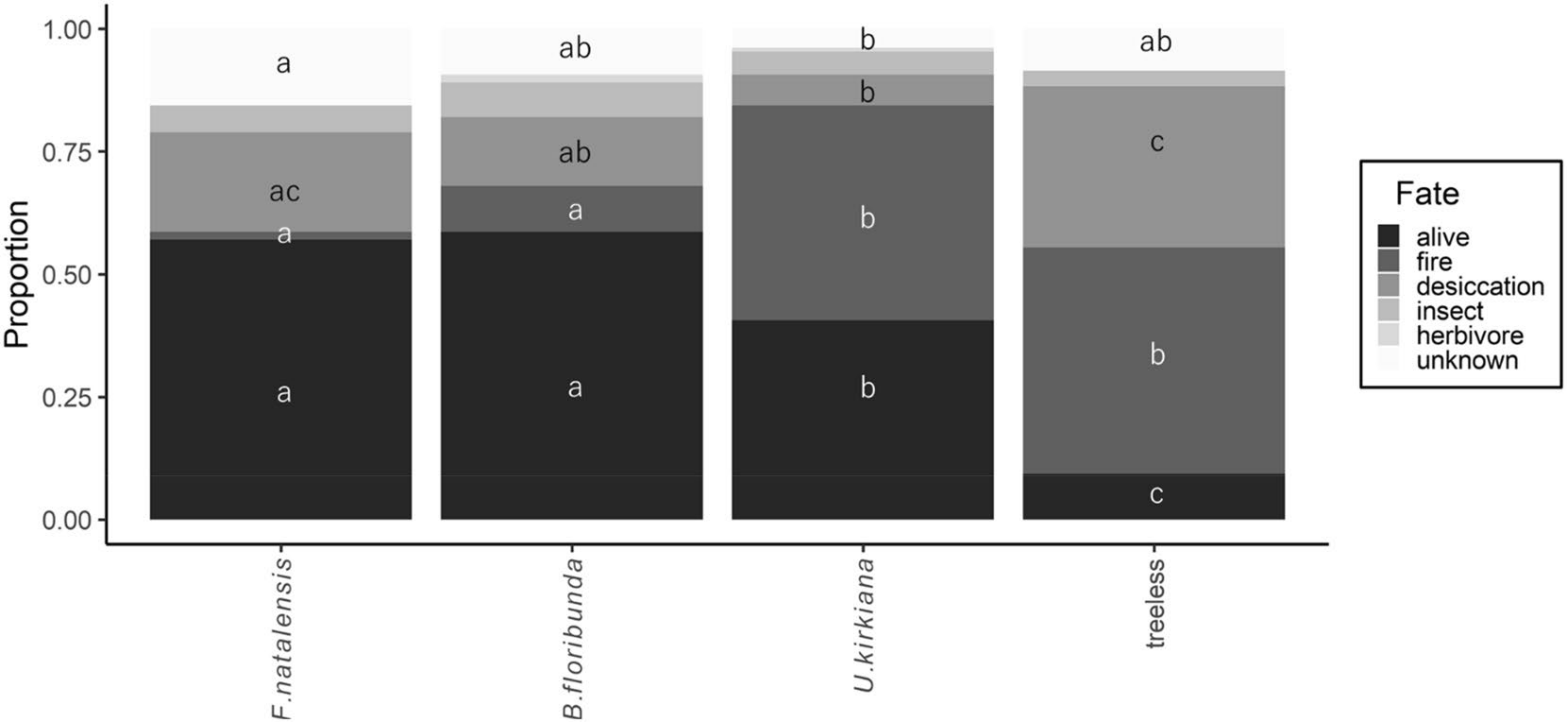
Fate of *Syzygium guineense* ssp. *afromontanum* transplants at the 4 microsites after 2.5 years. Seedling survival (n = 512) was measured from February 2012 to August 2014. All microsites were located in miombo woodlands. Different letters within each fate class indicate significant differences (*P* < 0.05) based on Tukey’s post hoc tests.

Overall, the seedling survival rate of *S. guineense* ssp. *afromontanum* after 2.5 years was 41%; however, rates differed among microsites. Survival was higher and similar under *F*. *natalensis* and *B*. *floribunda* than under *U*. *kirkian*a and at the treeless sites (Fig. 2). Causes of mortality were determined for 84% of dead seedlings at the end of the experiment. Fire was the most important source of mortality (43%), and was higher under *U*. *kirkiana* (74%) and at the treeless sites (51%). No differences were observed in fire-induced mortality between the *F. natalensis* (4%) and *B. floribunda* microsites (23%). Desiccation was the second most important source of mortality (31%) and was greatest at the treeless site. Insect damage (9%) and trampling by ungulates (1%) resulted in fewer deaths, and both factors had similar effects at all microsites.

**Figure 3:**
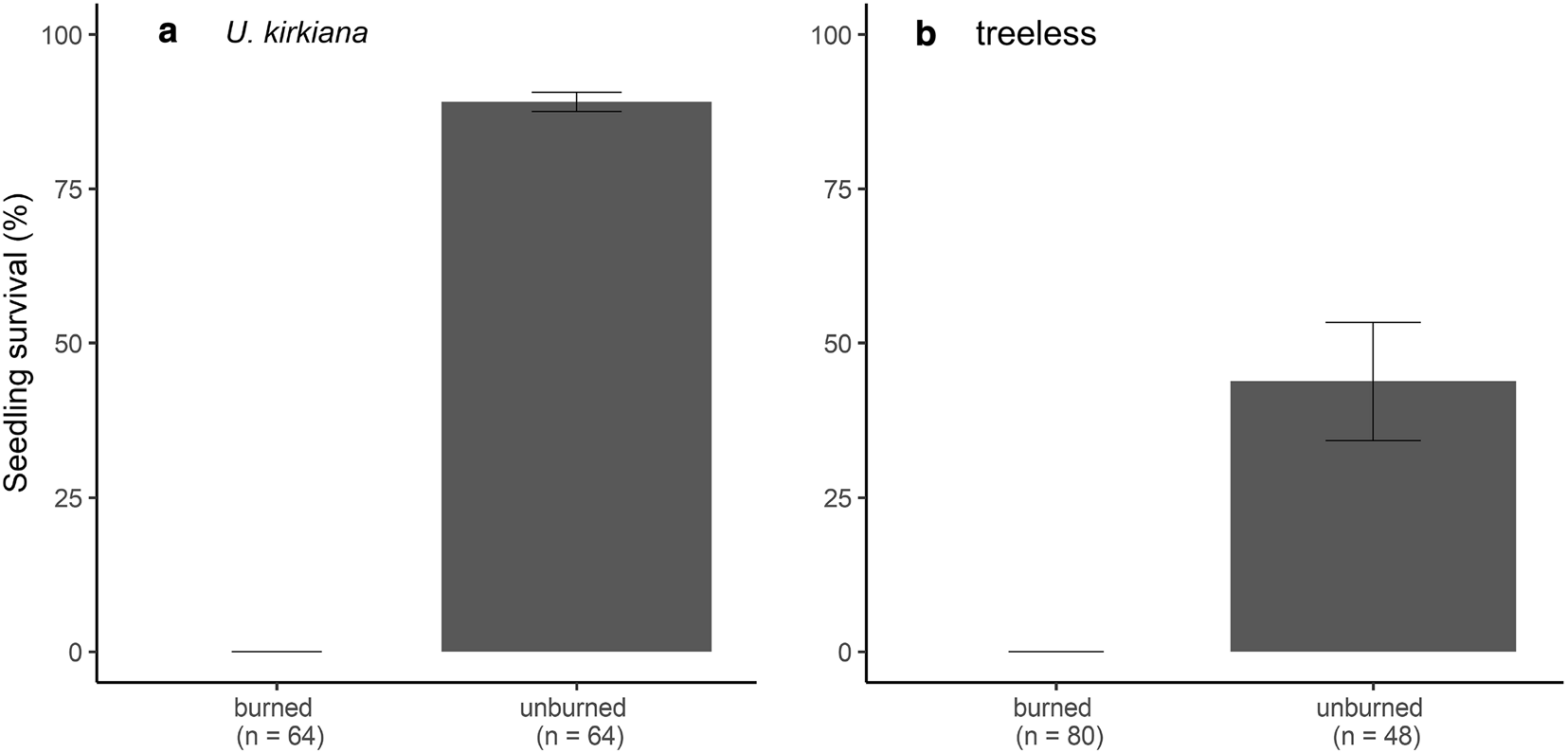
Comparisons of mean seedling survival (± 1 SE) between sites where fire occurred and those where it did not.

Fire burned only 1 out of 8 replicates for the *F*. *natalensis* and *B*. *floribunda* microsites. In contrast, fires occurred in 4 out of 8 replicates for the *U*. *kirkiana* microsites and 5 out of 8 replicates for treeless microsites. Survival under *U. kirkiana* (Fig. 3a) and at the treeless microsites (Fig. 3b) was subsequently compared between those sites in which fire occurred and those in which it did not. The results revealed relatively higher survival in sites where no fire occurred (89% under *U. kirkiana*, 44% at the treeless microsites). No seedlings survived in sites where fires occurred (Fig. 3).

### Natural establishment of *S. guineense* ssp. *afromontanum* and other forest tree species in miombo woodland

The ground surface of all four square sites was mostly treeless (51.6%), followed by *B. floribunda* (18.7%), *B. boehmii* (11.1%) and *U. kirkiana* (8.4%) (Table 1). Only 2.9% of the ground surface was occupied by *F. natalensis*. Overall, 285 *S. guineense* ssp. *afromontanum* seedlings and 186 (11 species) seedlings of other forest tree species (Table S1) were found in the four sites. Of the 285 *S. guineense* ssp. *afromontanum* seedlings, 146 were found under *F. natalensis*. Similarly, 135 of the additional 186 forest tree species seedlings were also found under *F. natalensis*. Overall, both the *S. guineense* ssp. *afromontanum* and other forest tree seedlings were more closely associated with *F. natalensis* than expected according to crown area. All individuals were checked for damage from herbivorous mammals, with only a few individuals showing damage (15 *S. guineense* ssp. *afromontanum* seedlings and 15 individuals of other forest tree species).

**Table 1:** Spatial association between microsites and *Syzygium guineense* ssp. *afromontanum* and other forest species seedling in miombo woodland in northern Malawi.

Microsite	Proportion of ground cover (%)	Observed number of *S*. *guineense* ssp. *afromontanum* seedling	Expected number of *S*. *guineense* ssp. *afromontanum* seedling	Observed number of other forest species seedling	Expected number of other forest species seedling
Treeless microsite	51.6	3	147.0	1	96.0
*Brachystegia floribunda*	18.7	25	53.3	9	34.8
*Brachystegia boehmii*	11.1	34	31.5	17	20.6
*Uapaka kirkiana*	8.4	41	24.1	1	15.7
*Monotes africana*	3.2	16	9.2	0	6.0
*Ficus natalensis*	2.9	146	8.1	135	5.3
Other species	4.1	20	11.7	23	7.6
Total number of the seedling observed	–	285	–	186	–

### Seed deposition of *S. guineense* ssp. *afromontanum*

**Figure 4:**
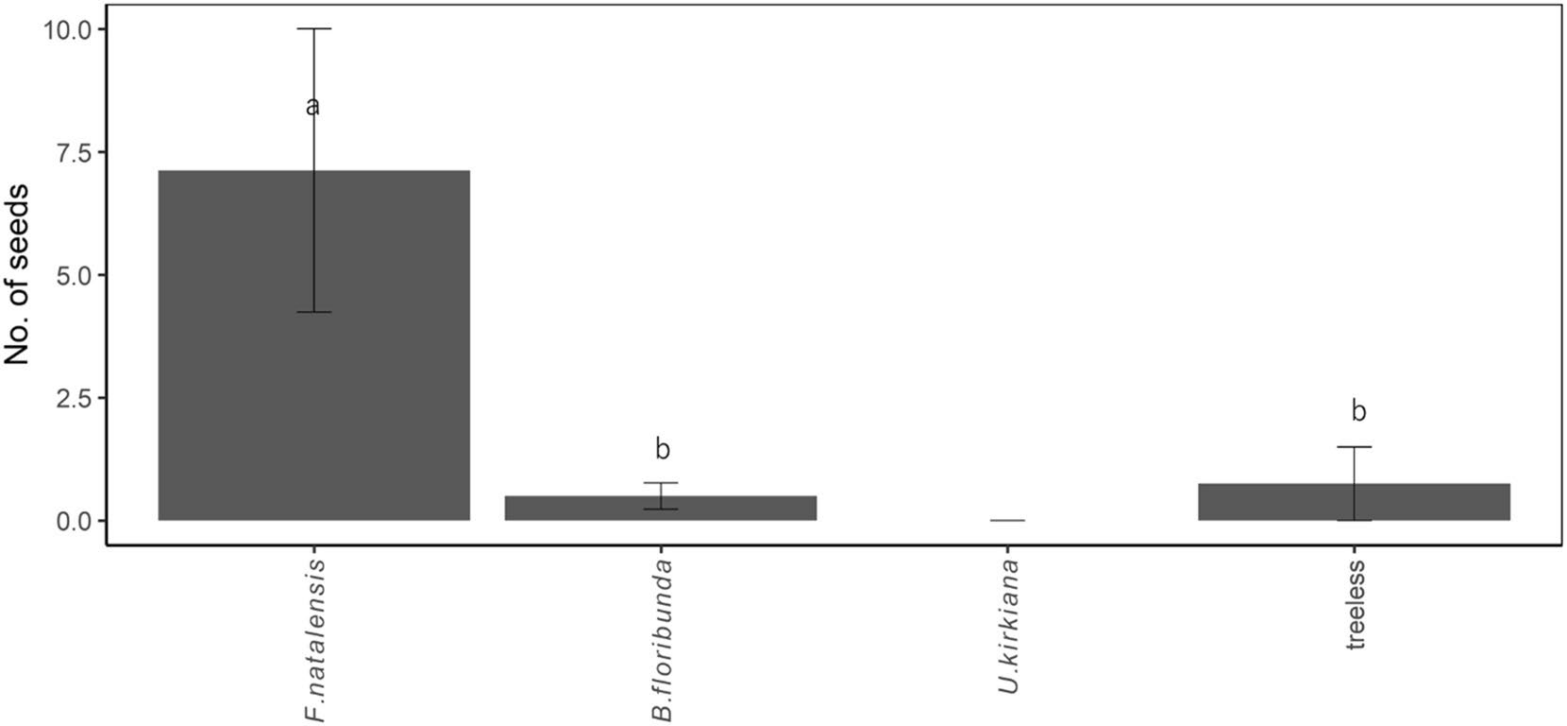
Mean number of dispersed seeds of *Syzygium guineense* ssp. *afromontanum* (± 1 SE) at 4 microsites in northern Malawi. All microsites were located in miombo woodlands. Seed rain was monitored from January to March 2012 using three seed traps at each of the four microsites (n = 96). Different letters within each fate class indicate significant differences (*P* < 0.05) based on Tukey’s post hoc tests.

The seed deposition of *S. guineense* ssp. *afromontanum* varied among the four microsites (Fig. 4). No seed was observed under *U*. *kirkiana* and the deposition data from these sites were excluded from the subsequent analysis. Most (85%) of the seeds were deposited under *F. natalensis*, with significantly more seeds deposited under *F. natalensis* than other microsites.

## Discussion

Protection from fire appears to be the most important facilitation mechanism affecting increased survival of forest tree species in miombo woodland in northern Malawi. Overall, fire was the cause of 43% of *S. guineense* ssp. *afromontanum* seedling deaths, suggesting that fire is the principal driver of seedling mortality (Fig. 2). The majority (89%) of deaths occurred at the treeless sites and under *U. kirkiana*, where grass cover was high, thereby increasing the fuel load. In contrast, fire-induced mortality was very low under *F. natalensis* and under *B. floribunda* (4 and 23%, respectively). In both microsites, fire is thought to be inhibited due to the closed crown, which prevents light-demanding C4 grasses from becoming dominant^30^. This effect, combined with the changes in microclimatic conditions provided by the closed crown, drastically decrease overall flammability. Because the intervals between seedling monitoring were long, I may have overestimated the effect of fire on seedling mortality. I cannot rule out the possibility that seedings were dead because of factors other than fire, but fire burned the dead individual after the seedling death. Furthermore, seedling survival at the treeless microsite and under *U*. *kirkiana* in which fire did not occur was relatively high (Fig. 3). These results suggest that *S*. *guineense* ssp. *afromontanum* seedlings can survive in miombo woodland in the absence of fire.

Drought was the second most important factor of the seedling mortality (31% of deaths), but fewer individuals under *F. natalensis* and *B. floribunda* were killed by drought compared with treeless microsites (Fig. 2). It was previously suggested that protection from drought plays a key facilitative role in many stressful ecosystems^31–33^. Further long-term studies are now required to determine the relative importance of fire and drought on the mortality of forest tree species during forest expansion.

No seedlings died due to damage by vertebrate herbivory during the 2.5-year study period, suggesting that vertebrate herbivores are not an important factor in seedling mortality in miombo woodland. Rao et al.^34^ suggested that although vertebrate herbivores have limited impact on seedlings at an early stage, they become more critical at later stages when seedlings grow taller and become more visible to ungulates. However, the observations of natural establishment in this study revealed only a few individuals with grazing damage, suggesting that vertebrate herbivores have a limited impact on the establishment of forest tree species in this region, even at later stages. This was surprising given that herbivorous mammals, along with fire, pose a major constraint on tree establishment in African savannah-woodland^35,36^. Although the reason for my finding is unknown, it may be attributed to the decrease in herbivores resulting from human impacts such as hunting^37,38^.

Soil-related factors might also be important determinants of seedling establishment and survival^39^. Soil fertility is generally higher in tropical forest than in adjacent savannah-woodland^40,41^, and some studies have suggested that the nutrient-poor soil of savannah-woodlands limits the establishment of forest tree species, thereby preventing forest development. Thus, it is possible that the successful seedling survival observed under *F. natalensis* and *B. floribunda* was due to the facilitative effect of increased soil nutrient availability. Increased soil nutrients under nurse plants is a widely known mechanism of facilitation^42^. Future studies are needed to examine this further and fully understand the facilitative effects on the establishment of forest tree species.

The findings also suggest that *B. floribunda* offers a similar degree of protection from fire as *F. natalensis*. In fact, seedling mortality due to fire and seedling survival did not differ between the *B. floribunda* and *F. natalensis* microsites (Fig. 2). However, natural establishment of *S. guineense* ssp. *afromontanum* and other forest tree species tended to be concentrated under *F. natalensis*, with few seedlings under *B. floribunda* (Table 1). These results suggest that the facilitative mechanism of fire suppression does not fully explain the recruitment of forest tree species in the miombo woodland, indicating the involvement of other processes. Here, the pattern of seed deposition by frugivorous animals is likely critical for the recruitment of forest tree species^18,20,22,43–45^. In this study, higher seed deposition of *S. guineense* ssp. *afromontanum* was observed under *F. natalensis* than in the other three microsites. Moreover, the forest tree species observed under *F. natalensis* were largely animal dispersed, further supporting this hypothesis.

In conclusion, this study highlights the potential mechanisms of forest tree species establishment in miombo woodland. The findings suggest that ground cover beneath the closed crown of *F. natalensis* is less likely to be burned, thus increasing survival of *S. guineense* ssp. *afromontanum* seedlings. Fire suppression is very important because forest tree species are generally very vulnerable to fire^8,10,11^. Previous empirical and theoretical studies on forest-savannah dynamics further suggest the importance of fire suppression on forest expansion into adjacent savannah-woodland^3,46^. However, the present study also suggests that fire suppression is not the only factor affecting the establishment of forest tree species. Fire is also unlikely to occur under the closed crown of *B. floribunda* (Fig. 2) but few forest tree species were naturally established at these microsites (Table 1). Overall, these findings suggest that in addition to fire suppression, dispersal limitations also play a role in forest-savannah dynamics in this region, especially at the community level.

## Supplementary Information

Supplementary Information.

## References

[1] Mitchard ETA, Saatchi SS, Gerard FF, Lewis SL, Meir P (2009). Measuring woody encroachment along a forest-savanna boundary in Central Africa. Earth Interact..

[2] Murphy BP, Bowman DMJS (2012). What controls the distribution of tropical forest and savanna?. Ecol. Lett..

[3] Staver AC, Archibald S, Levin S (2011). Tree cover in sub-Saharan Africa: Rainfall and fire constrain forest and savanna as alternative stable states. Ecology.

[4] Bowman DMJS, Murphy BP, Banfai DS (2010). Has global environmental change caused monsoon rainforests to expand in the Australian monsoon tropics?. Landsc. Ecol..

[5] Favier C, Namur CD, Dubois MF (2004). Forest progression modes in littoral Congo, central atlantic Africa. J. Biogeogr..

[6] Puyravaud JP, Dufour C, Aravajy S (2003). Rain forest expansion mediated by successional processes in vegetation thickets in the Western Ghats of India. J. Biogeogr..

[7] Tng DYP (2012). Humid tropical rain forest has expanded into eucalypt forest and savanna over the last 50 years. Ecol. Evol..

[8] Mariano V, Rebolo IF, Christianini AV (2019). Fire sensitive species dominate seed rain after fire supression: implications for plant community diversity and woody encroachment in the Cerrado. Biotropica.

[9] Ferreira AV, Bruna EM, Vasconcelos HL (2010). Seed predators limit plant recruitment in neotropical savannas. Oikos.

[10] Azihou AF, Glèlè Kakaï R, Sinsin B (2013). Do isolated gallery-forest trees facilitate recruitment of forest seedlings and saplings in savannna?. Acta Oecol..

[11] Duarte LDS, Dos-Santos MMG, Hartz SM, Pillar VD (2006). Role of nurse plants in Araucaria Forest expansion over grassland in south Brazil. Austral Ecol..

[12] Hoffmann WA (1996). The Effects of Fire and Cover on Seedling Establishment in a Neotropical Savanna. J. Ecol..

[13] Hoffmann WA, Orthen B, Franco AC (2004). Constraints to seedling success of savanna and forest trees across the savanna-forest boundary. Oecologia.

[14] Lawes MJ, Murphy BP, Midgley JJ, Russell-Smith J (2011). Are the eucalypt and non-eucalypt components of Australian tropical savannas independent?. Oecologia.

[15] Russell-Smith J, Stanton PJ, Whitehead PJ, Edwards A (2004). Rain forest invasion of eucalypt-dominated woodland savanna, iron range, north-eastern Australia: I. Successional processes. J. Biogeogr..

[16] Bruno JF, Stachowicz JJ, Bertness MD (2003). Inclusion of facilitation into ecological theory. Trends Ecol. Evol..

[17] Callaway RM (1995). Positive interactions among plants. Bot. Rev..

[18] Slocum MG, Horvitz CC (2000). Seed arrival under different genera of trees in a neotropical pasture. Plant Ecol..

[19] Schlawin JR, Zahawi RA (2008). ‘Nucleating’ succession in recovering neotropical wet forests: the legacy of remnant trees. J. Veg. Sci..

[20] Slocum MG (2001). How tree species differ as recruitment foci in a tropical pasture. Ecology.

[21] Fujita T (2014). Ficus natalensis facilitates the establishment of a montane rain-forest tree in south-east African tropical woodlands. J. Trop. Ecol..

[22] de Dantas VL (2007). Plant dispersal strategies and the colonization of araucaria forest patches in a grassland-forest mosaic. J. Veg. Sci..

[23] Campbell B, Frost P, Byron N, Campbell B (1996). Miombo woodlands and their use: overview and key issues. The Miombo in transition: woodlands and welfare in Africa.

[24] White F, Dowsett-Lemaire F, Chapman S (2001). Evergreen Forest Flora of Malawi.

[25] Chapman JD, Chapman JD, White F (1970). PART II Description of the forest. The evergreen forests of Malawi.

[26] Hoffmann WA (2012). Ecological thresholds at the savanna-forest boundary: how plant traits, resources and fire govern the distribution of tropical biomes. Ecol. Lett..

[27] Frazer, G. W., Canham, C. D., & Lertzman, K. P. Gap Light Analyzer (GLA) 2.0: Imaging software to extract canopy structure and gap light transmission indices from true-colour fisheye photographs. http://remmain.rem.sfu.ca/downloads/Forestry/GLAV2UsersManual.pdf (1999).

[28] Bates D, Mächler M, Bolker B, Walker S (2015). Fitting linear mixed-effects models using lme4. J. Stat. Softw..

[29] Hothorn T, Bretz F, Westfall P (2008). Simultaneous inference in general parametric models. Biom. J..

[30] Trauernicht C, Murphy BP, Portner TE, Bowman DMJS (2012). Tree cover-fire interactions promote the persistence of a fire-sensitive conifer in a highly flammable savanna. J. Ecol..

[31] Castro J, Zamora R, Hódar JA, Gómez JM (2004). Seedling establishment of a boreal tree species (*Pinus sylvestris*) at its southernmost distribution limit: consequences of being in a marginal Mediterranean habitat. J. Ecol..

[32] Gómez-Aparicio L, Gómez JM, Zamora R, Boettinger JL (2005). Canopy vs. soil effects of shrubs facilitating tree seedlings in Mediterranean montane ecosystems. J. Veg. Sci..

[33] Smit C, Den Ouden J, Díaz M (2008). Facilitation of Quercus ilex recruitment by shrubs in Mediterranean open woodlands. J. Veg. Sci..

[34] Rao SJ, Iason GR, Hulbert IAR, Elston DA, Racey PA (2003). The effect of sapling density, heather height and season on browsing by mountain hares on birch. J. Appl. Ecol..

[35] de Dantas VL, Hirota M, Oliveira RS, Pausas JG (2016). Disturbance maintains alternative biome states. Ecol. Lett..

[36] Terborgh J (2016). Megafaunal influences on tree recruitment in African equatorial forests. Ecography.

[37] Ripple R (2016). Bushmeat hunting and extinction risk to the world's mammals. R. Soc. Open Sci..

[38] Hegerl C, Burgess N, Nielsen M, Martin E, Ciolli M, Rovero F (2017). Using camera trap data to assess the impact of bushmeat hunting on forest mammals in Tanzania. Oryx.

[39] Bowman DMJS, Panton WJ (1993). Factors that control monsoon-rainforest seedling establishment and growth in North Australian Eucalyptus Savanna. J. Ecol..

[40] Ruggiero CPG, Batalha MA, Pivello VR, Meirelles ST (2002). Soil-vegetation relationships in cerrado (*Brazilian savanna*) and semideciduous forest, Southeastern Brazil. Plant Ecol..

[41] Viani RAG, Rodrigues RR, Dawson TE, Oliveira RS (2011). Savanna soil fertility limits growth but not survival of tropical forest tree seedlings. Plant Soil.

[42] Chen J, Yang Y, Stöcklin J, Cavieres LA, Peng D, Li Z, Sun H (2015). Soil nutrient availability determines the facilitative effects of cushion plants on other plant species at high elevations in the south-eastern Himalayas. Plant Ecolog. Divers..

[43] Zahawi RA, Holl KD, Cole RJ, Reid JL (2013). Testing applied nucleation as a strategy to facilitate tropical forest recovery. J. Appl. Ecol..

[44] Clark CJ, Poulsen JR, Connor EF, Parker VT (2004). Fruiting trees as dispersal foci in a semi-deciduous tropical forest. Oecologia.

[45] Carlo TA, Aukema JE (2005). Female-directed dispersal and facilitation between a tropical mistletoe and a dioecious host. Ecology.

[46] Bond WJ, Woodward FI, Midgley GF (2005). The global distribution of ecosystems in a world without fire. New Phytol..

