# Supplementary material for: Fire suppression and seed dispersal play critical roles in the establishment of tropical forest tree species in southeastern Africa
Source: Sci Rep. 2021 Aug 12;11:16391. doi: 10.1038/s41598-021-95752-7 (PMC8361061; doi:10.1038/s41598-021-95752-7)
Supplement: Supplementary file 1 — Supplementary Information. [file 41598_2021_95752_MOESM1_ESM.docx]

**Fire suppression and seed dispersal play critical roles in the establishment of tropical forest tree species in southeastern Africa**

**Tomohiro Fujita^1,*^**

^1^ Center for Climate Change Adaption, National Institute for Environmental Studies, 16-2, Onogawa, Tsukuba, Ibaraki, 305-8506, Japan

**Appendix**

| Table S1. List of forest tree species seedling at three microsites in northern Malawi. | |  |  |  |  |
| --- | --- | --- | --- | --- | --- |
| Species name | Family | Fruit size (mm) | Fruit color | Mode of seed dispersal | Habitat |
|  |  |  |  |  |  |
| *Cussonia spicata* Thunb. ^a^ | Araliaceae | 7 × 7 | purple | animal | for./woodl. |
| *Schefflera umbellifera* (Sond.) Baill. ^a^ | Araliaceae | 3 in dia. | red | animal | forest |
| *Diospyros whyteana* (Hiern) F. White ^b^ | Ebenaceae | 20 × 20 | red | animal | forest |
| *Apodytes dimidiata* E. Mey. ex Arn. ^b^ | Icacinaceae | 10 in dia. | black | animal | forest |
| *Grewia stolzii* Ulbr. ^b^ | Malvaceae | 30 in dia. | unknown | animal | forest |
| *Bersama abyssinica* var. *engleriana* (Gürke) F. White ^b^ | Melianthaceae | 10 × 8 | red | animal | for./woodl. |
| *Schrebera alata* (Hochst.) Welw. ^b^ | Oleaceae | - | - | explosive | forest |
| *Prunus africana* (Hook. f.) Kalkman ^b^ | Rosaceae | 10 × 7 | brown | animal | forest |
| *Oxyanthus speciosus* spp. *stenocarpus* (K. Schum.) Bridson ^b^ | Rubiaceae | 30 in dia. | yellow | animal | forest |
| *Psydrax schimperiana* (A.Rich.) Bridson ^b^ | Rubiaceae | 10 in dia. | black | animal | forest |
| *Allophylus chaunostachys* Gilg ^b^ | Sapindaceae | 6 in dia. | red | animal | for./woodl. |
| for., forest; woodl., woodland; dia., diameter. |  |  |  |  |  |
| a: Coutes Palgrave (2002). b: White et al. 2001. |  |  |  |  |  |

Fig. S1


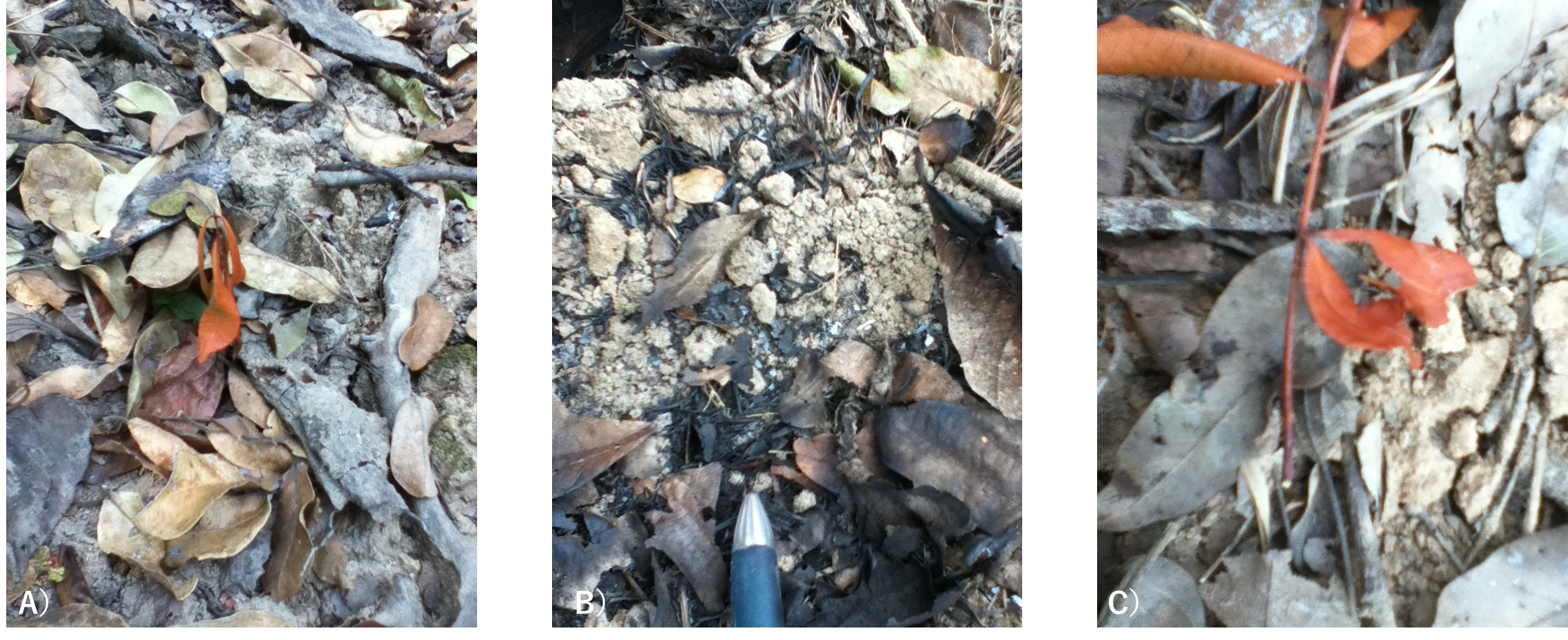


Photo of dead *S. guineense* ssp. *afromontanum* seedlings. The photo were taken by Tomohiro Fujita.
